# Modeling of senescent cell dynamics predicts a late‐life decrease in cancer incidence

**DOI:** 10.1111/eva.13514

**Published:** 2023-03-01

**Authors:** Margaux Bieuville, Tazzio Tissot, Alexandre Robert, Pierre‐Yves Henry, Samuel Pavard

**Affiliations:** ^1^ Eco‐Anthropologie (EA UMR 7206), MNHN, CNRS Université Paris‐Diderot Paris France; ^2^ Agent, Interaction and complexity (AIC) research group Southampton University Southampton UK; ^3^ Centre d'Ecologie et des Sciences de la Conservation (CESCO UMR 7204), MNHN, CNRS Sorbonne Université Paris France; ^4^ Mécanismes Adaptatifs et Evolution (MECADEV UMR 7179), MNHN, CNRS Brunoy France

**Keywords:** ageing, cancer, demography, evolution, life‐history traits, Peto's paradox, senescent cells

## Abstract

Current oncogenic theories state that tumors arise from cell lineages that sequentially accumulate (epi)mutations, progressively turning healthy cells into carcinogenic ones. While those models found some empirical support, they are little predictive of intraspecies age‐specific cancer incidence and of interspecies cancer prevalence. Notably, in humans and lab rodents, a deceleration (and sometimes decline) of cancer incidence rate has been found at old ages. Additionally, dominant theoretical models of oncogenesis predict that cancer risk should increase in large and/or long‐lived species, which is not supported by empirical data. Here, we explore the hypothesis that cellular senescence could explain those incongruent empirical patterns. More precisely, we hypothesize that there is a trade‐off between dying of cancer and of (other) ageing‐related causes. This trade‐off between organismal mortality components would be mediated, at the cellular scale, by the accumulation of senescent cells. In this framework, damaged cells can either undergo apoptosis or enter senescence. Apoptotic cells lead to compensatory proliferation, associated with an excess risk of cancer, whereas senescent cell accumulation leads to ageing‐related mortality. To test our framework, we build a deterministic model that first describes how cells get damaged, undergo apoptosis, or enter senescence. We then translate those cellular dynamics into a compound organismal survival metric also integrating life‐history traits. We address four different questions linked to our framework: can cellular senescence be adaptive, do the predictions of our model reflect epidemiological patterns observed among mammal species, what is the effect of species sizes on those answers, and what happens when senescent cells are removed? Importantly, we find that cellular senescence can optimize lifetime reproductive success. Moreover, we find that life‐history traits play an important role in shaping the cellular trade‐offs. Overall, we demonstrate that integrating cellular biology knowledge with eco‐evolutionary principles is crucial to solve parts of the cancer puzzle.

## 1 INTRODUCTION

Cancer is currently the second leading cause of death in humans, and one of the major causes of disability worldwide (Bray et al., 2021; Sung et al., 2021). Massive investments in molecular and cellular research on the drivers of cancer initiation and progression have allowed to reduce the mortality of cancer patients over the years. Yet, the epidemiology of cancer and the factors underlying cancer prevalence, between species, and within and between populations of the same species, are still poorly understood.

Dominant theories of oncogenesis state that cancer is a multistage process, fueled by the accumulation of genetic and epigenetic alterations (also known as the Doll‐Armitage model of carcinogenesis, Doll & Armitage, 1954; Tomasetti & Vogelstein, 2015) that, in turn, confer pathological features to cancer cells (Hanahan & Weinberg, 2011). These alterations are accumulated randomly and sequentially by a somatic lineage of mitotically active cells over the course of divisions. Malignancy should only appear once the sequence of alterations is complete. This molecular model has been empirically verified many times and describes well how healthy cells turn into cancer cells after having accumulated about 4–10 mutations under positive selection (Martincorena et al., 2017). It is, however, little predictive of patterns of cancer prevalence and age‐specific incidence at the population/species level.

Indeed, this multistage carcinogenesis model leads to several predictions about such patterns. (i) Long‐lived animal species should have a higher cancer risk than short‐lived ones, as their cell lineages divide more times over their lives. (ii) Large animal species should have more cancers, as more cell lineages can independently undergo alterations. (iii) In any population, cancer incidence should increase steadily with age (but see different interpretations between Moolgavkar (2004) and Ritter et al. (2003)), as cell lineages may only accumulate more mutations. Though plausible under the multistage carcinogenesis model, these expectations are actually little supported by empirical data.

On the one hand, no positive statistical link has yet been found among mammals between a species' cancer risk and either its body size or its lifespan (Vincze et al., 2022), a phenomenon known as Peto's paradox (Nunney, 1999). Rather, cancer risk seems to be shaped by phylogeny and ecology (Madsen et al., 2017; Vincze et al., 2022).

On the other hand, for most cancers in humans (but also rats, see Anisimov et al. (2004), reviewed by Anisimov et al. (2005) and domestic dogs (Dobson et al., 2002), incidence does increase with age; but only until a point where incidence increase decelerates and incidence may even decline at old ages. For instance, in humans, cancer incidence tends to decrease past ages 75–90 years (Nolen et al., 2017), making cancer one of the least prevalent causes of death in centenarians (Pavlidis et al., 2012). Such deceleration/decline late in adult life has been known for the last 50 years (Cook et al., 1969), and several hypotheses have been proposed to explain this pattern as a population bias in incidence calculation (reviewed in Anisimov et al. (2005). However, all these hypotheses are so far insufficient to explain this epidemiological paradox. (i) A *detection bias* assumes that the oldest patients are the most difficult to screen for tumors with invasive procedures. But this bias should have been reduced with improvements in less invasive imaging technology. (ii) A *cohort‐period bias* assumes that the oldest patients do have less cancers because they lived in less carcinogenic environments. Yet the pattern still holds when controlling for cohort and period effects (Hanson et al., 2015). (iii) A *survival bias* assumes that due to environmental and genetic heterogeneity within a population, people have unequal chances to develop cancer (due to genetic susceptibility, environment, or behaviors) so that the selective disappearance of the most cancer‐prone individuals only leaves alive the least cancer‐prone individuals at the oldest ages (Vaupel & Yashin, 1986). However, so far, there has been little empirical data on humans in support (or in contradiction) of this assumption (discussed in Pavard and Metcalf (in press)). Furthermore, in rodents, a late‐life decrease in cancer incidence could still be observed even when removing most of the environmental factors (by using controlled environments) and genetic heterogeneity (by using isogenic lineages; Anisimov et al., 2005). An increasing body of research therefore deems this pattern, not only as a population bias in incidence calculation but also as a physiological mechanism in which somatic ageing may reduce the incidence of cancer with age (Ukraintseva & Yashin, 2001, 2003), e.g., through the decrease in cell divisions with age (Tomasetti et al., 2019) or cellular replicative senescence (Anisimov et al., 2005).

The aim of this study is to explore whether cellular senescence (henceforth, CS) could explain this population and species pattern of cancer prevalence and incidence decrease with age. Indeed, if carcinogenesis confers to cell lineages the ability to perform mitosis indefinitely and uncontrollably, cellular senescence is contrariwise defined by a stable arrest of the cell cycle (Campisi & d'Adda di Fagagna, 2007). These two pathways seem to be antagonistic in their effects but also in their causes. On the one hand, the entry into CS is generally triggered by either telomere attrition over mitotic divisions, or the p53 and p16‐pRB pathways in case of nontelomeric DNA damage (Ibid). On the other hand, most cancers cannot thrive without fighting telomere attrition by the overexpression of telomerase (Blasco, 2005), and loss‐of‐function mutations in the genes p53, p16, and pRb desensitize cancer cells to apoptosis and senescence (Sherr & McCormick, 2002). An organism favoring the entrance of damaged cells into senescence should therefore suffer from reduced levels of carcinogenesis. Thus, many authors have suggested that CS could be an adaptation against cancer (Campisi, 2001, 2005; Campisi & d'Adda di Fagagna, 2007; Finkel et al., 2007; He & Sharpless, 2017; van Deursen, 2014).

Whether CS can be adaptive is, however, so far unknown. Besides, increasing evidence also shows that the accumulation of senescent cells in tissues is one of the causes of actuarial ageing and ultimately of body impairment, at least at old ages (Baker et al., 2011; He & Sharpless, 2017; López‐Otín et al., 2013; van Deursen, 2014). Therefore, a new field of pharmacology is currently investigating whether chemical components triggering apoptosis in (otherwise apoptosis‐resistant) senescent cells (i.e., senolysis) could, at least, alleviate ageing symptoms if not rejuvenate tissues. In progeroid mice, (Baker et al., 2011) showed that clearance of senescent cells delayed the onset of ageing‐related disorders. Later, in normally‐aging mice, the same group of researchers showed that senolysis also delayed tumorigenesis and extended the median lifespan of the animals (Baker et al., 2016).

From these two effects should therefore emerge a trade‐off between mortality components where organisms would trade one cause of death (cancer) against another ones (ageing‐related; Pavard & Metcalf, in press). Such trade‐off, mediated by the accumulation of senescent cells in tissues with age, could theoretically be adaptive under a basic set of assumptions and could lead to a late‐life decrease in cancer incidence (Pavard & Metcalf, in press).

But CS is not the only cell‐autonomous pathway that can prevent carcinogenesis: apoptosis (programmed cell death) could eliminate damaged cells from the tissues indefinitely if there were no physiological cost to doing so. However, apoptosis also leads to the replacement of damaged cells through mitosis of the remaining cells. These latter compensatory divisions (Pérez‐Garijo, 2018) do not lead to tissue ageing, but could theoretically lead to (i) additional mutations and therefore to higher levels of damage accumulation and (ii) to a faster stem cell exhaustion. Thus, apoptosis would maintain short‐term organism integrity but potentially at the cost of a delayed higher cancer risk. Both apoptosis and CS are therefore expected to delay the mortality of organisms and increase their life expectancy. However, they favor the onset of different mortality components differently: apoptosis should increase cancer risk, and CS should favor ageing‐related causes of death.

In this study, we build a toy model to explore the effect of such a trade‐off. More precisely, we model the dynamics of damaged and senescent cells with age in tissues, where damaged cells can undergo apoptosis (and lead to compensatory divisions) or enter into senescence. A trade‐off occurs at the organism level because the proportion of damaged cells increases the risk of death by cancer while that of senescent cells increases the risk of other ageing‐related death. The model makes explicit the longevity of cell lines. It models at the tissue level the costs (i) of the accumulation of senescent cells in terms of additional damage probability due to tissue deterioration and (ii) of apoptosis in terms of damage emergence due to compensatory divisions. It then models, at the organism level, the resilience of the organism to senescent and damaged cell accumulation in terms of organism survival. At the organism level, it also makes explicit life‐history traits such as extrinsic mortality and senescence of reproduction. It is important to note that this model is derived from the Doll‐Armitage multistage model where the age‐specific risk of carcinogenesis is solely determined by the number of accumulated “hits.”

With this model in hand we address several questions: (i) Can we find parameter combinations where the accumulation of senescent cells increases the organism's Lifetime Reproductive Success (hereafter, “*LRS*”)? If so, what are the drivers of such an increase and their influence on the lifetime cancer prevalence? (ii) Do these parameter combinations lead to the deceleration and decline of cancer incidence with age? (iii) Can we predict the effect that increasing body size should have on the lifetime cancer prevalence? (iv) In case of a senolytic experiment (killing senescent cells at a given age), can we predict its effect on the lifetime cancer prevalence and organism longevity? Are those results congruent with the in vivo models?

## 2 METHODS

This model is a generalization of the one published by Pavard and Metcalf (in press) that modeled senescent and damaged cell dynamics and their consequent effects on organism morbidity. Our model now includes apoptosis as an alternative mechanism preventing damage accumulation in cell lineages, as well as life‐history parameters.

### 2.1 Description of the model

We first modeled dynamics of cells in the organism. We considered an organism formed of a single tissue, in which three types of cells can coexist: healthy cells, damaged cells, and senescent cells. Let us denote *S*_*t*_ and *D*_*t*_, respectively, the proportions of senescent and damaged cells and 1‐*S*_*t*_ *‐D*_*t*_ the proportion of undamaged mitotic cells. During the time step *t*, a proportion *α*_*t*_ of normal cells turns into damaged cells. Parameter *α*_*t*_ accounts for the age‐specific rate at which healthy cells get damaged by the unit of time and therefore aggregates the rate at which healthy cells divide, get damaged, and stay unrepaired (see Table 1). Here we modeled an increasing genetic instability with age by considering a linear increase of this rate such that *α*_*t*_ = αt setting *α*_*t*_ = *1* for *t* > 1/*α*. To account for potential costs of CS in terms of damage accumulation due to impaired tissue functioning (e.g., arising from inflammation phenotype or loss in tissue connectivity), an additional proportion of damaged cells *α*_*t*_ *S*_*t*_^rDamageToCS^ was generated at each time step as a function of the proportion of senescent cells, where parameter rDamageToCS is the tissue resilience to loss of tissue function resulting from the accumulation of senescent cells. At a time step *t*, the proportion *δ*_*t*_ of healthy cells getting damaged could therefore be written as:

(1) $$\delta_{t}=\left(1-S_{t}-D_{t}\right)\;\alpha_{t}\left(1+{S_{t}}^{\text{rDamageToCS}}\right)$$

**TABLE 1:** Parameters and derived metrics of the model

Input parameters			
Mechanism	Notation	Description	Biological meaning
Transition from healthy to damaged cell	*α*	Sets the probability *α*_*t*_ = α.*t* that healthy cells turn damaged at time *t*	Aggregates rate of cell division per time step, mutation rate, and repair efficiency. Therefore, for a given rate of cell division per time step, it accounts for increasing genomic instability with age
	rDamageToC*S*	Sets a power function between the proportion of senescent cells that have accumulated in tissue and the probability that cells get damaged	Describes the extent to which senescent cells may compromise tissue function through loss of tissues' connectivity or increased inflammation, which may in turn increase the damage rates of dividing cells
Fate of a damaged cell	*σ*	Probability that a damaged cell enters into senescence	Sets CS efficiency to prevent damaged cells to turn into cancer but at the cost of senescent cell accumulation during the organism's life
	*γ*	Probability that a damaged cell undergoes apoptosis	Sets apoptosis efficiency to prevent damaged cells to turn into cancer but at the cost of *compensatory proliferation*
Mortality by cancer and senescence	rCancerToDC	Sets a power function between the proportion of damaged cells and cancer survival	Describes the resilience to cancer of an organism that accumulated a given proportion of damaged cells
	rSenescToCS	Sets a power function between the proportion of senescent cells and survival to (other than cancer) ageing‐related causes of death	Describes the resilience to (other than cancer) senescent‐related disorders of an organism that accumulated a given proportion of senescent cells
Life‐history	extM	Sets the level of mortality per time step independently of age	Models the level of irreducible *in natura* environmental deaths (e.g., coming from predation, infection, or accidents)
	SenescRepro	Sets the rate at which reproduction efficiency declines with age	Models a potential senescence of reproduction with age
Derived metrics properties			
Cell lines undamaged life expectancy	$e_{0}^{\mathrm{cl}}$ (*α, γ*)	Mean number of time steps that a cell line will stay undamaged in absence of accumulation of senescent cells and organism death	Describes the potential longevity of cell lines with increased genomic instability with age *α*_*t*_, when only apoptosis removes damaged cells with a probability *γ*. This metric accounts for potential interaction between *α* and *γ*
Cancer‐only life expectancy	$e_{0}^{C}$ (*α, γ*, rCancerToDC)	Mean number of time steps that an organism will survive cancer in the absence of accumulation of senescent cells and extrinsic mortality	This metric accounts for potential interaction between *α*, *γ*, and rCancerToDC
Delta of resilience	*Δr*	Equals rSenescToCS‐rCancerToDC	Describes the sign and magnitude of the difference in survival costs resulting, respectively, from the accumulation of senescent cells and damaged cells
Extrinsic‐mortality‐only life expectancy	$e_{0}^{E}$	Equals 1/extM	Describe the life expectancy in the case where all deaths arise from extrinsic causes

Damaged cells could still potentially divide but increasing levels of damage (i.e., the accumulation of mutations along a cell lineage) were not considered here. We further considered that, over a time step, all damaged cells in the tissue (thus the previously damaged cells plus the newly damaged cells *D*_*t*_ + *δ*_*t*_) could undergo apoptosis with a probability *γ* and enter senescence with a probability *σ*. Cells are first checked for apoptosis and then for entry into senescence. Thus, the proportion of damaged cells undergoing apoptosis is *γ*, those entering senescence is (1‐*γ*) *σ*, and those that will remain damaged is (1‐*γ*) (1‐*σ*).

However, to maintain homeostasis, we hypothesize that cells undergoing apoptosis have to be replaced through compensatory divisions (see support for this hypothesis in, He and Sharpless (2017)). They can be replaced by the division of healthy cells or already damaged cells. Because our model is not spatially structured, the distribution of damage across the organism was assumed random. Then, the probability that a cell undergoing apoptosis was replaced by an already damaged cell depended on the proportion of damaged cells *D*_*t*_ over the proportion of potentially dividing cells (1‐*S*_*t*_). For simplicity, we did not consider new damage occurring during compensatory divisions because this rate is of an order of (*α*_*t*_)^2^ and is therefore negligible with respect to *α*_*t*_.

Therefore, the proportion of damaged cells *D*_*t+*1_ was the number of already and newly damaged cells, minus those entering into apoptosis, minus those entering into senescence, plus, among those entering into apoptosis, those that were replaced by division of damaged cells:

$$D_{t+1}=D_{t}+\delta_{t}-\left(D_{t}+\delta_{t}\right)\;\gamma -\left(D_{t}+\delta_{t}\right)\;\left(1-\gamma \right)\;\sigma +\left(D_{t}+\delta_{t}\right)\;\gamma \frac{D_{t}}{1-S_{t}},$$

which can be simplified by:

(2) $$D_{t+1}=\left(D_{t}+\delta_{t}\right)\;\left(\left(1-\gamma \right)\left(1-\sigma \right)+\gamma \frac{D_{t}}{1-S_{t}}\right)$$

The proportion of senescent cells *S*_*t+*1_ is:

(3) $$S_{t+1}=S_{t}+\left(D_{t}+\delta_{t}\right)\;\left(1-\gamma \right)\;\sigma$$

In the following, we are interested in modeling damage accumulation within tissues in fixed‐size, reproducing organisms, that is only during adult life. We therefore considered that age 1 is that of growth cessation and first reproduction (so that we model determinate growth species only). We further considered that organisms have no senescent or damaged cells at this age (i.e., *S*_*0*_ = *D*_*0*_ = 0), which we know is largely false since most damages occur during ontogenesis (DeGregori, 2013). These are, however, reasonable assumptions in (and only in) a Doll‐Armitage framework where an increased incidence of cancer in adult life depends on additional accumulated damage since maturity.

Once the dynamics of cells were modeled, we turned to their effects at the organism level. Organisms could die from cancer or other ageing‐related causes of death or from extrinsic mortality. We assumed that a high proportion of damaged cells was associated with a likely onset of cancer, so that the probability that an organism survives cancer at age *t* (denoted $p_{t}^{C}$) was negatively correlated with the proportion of damaged cells:

(4) $$p_{t}^{C}=1-{\left(D_{t}\right)}^{\text{rCancerToDC}}$$

where rCancerToDC is the organism's resilience to cancer due to the accumulation of damaged cells (see Table 1). Given a similar proportion, larger organisms are expected to exhibit a larger number of damaged cell lines. Therefore, parameter rCancerToDC also models the extent to which body size can impact cancer incidence (in absence of alternative mechanisms also affecting this relationship, such as the efficiency of the immune system to predate damaged cells turning into cancer).

Similarly, we assumed that a high proportion of senescent cells was associated with impaired tissue function leading to (other than cancer) ageing‐associated morbidity, so that the probability that an organism survives ageing at age *t* (denoted $p_{t}^{S}$) was negatively correlated with the proportion of senescent cells:

(5) $$p_{t}^{S}=1-{\left(S_{t}\right)}^{\text{rSenescToCS}}$$

where rSenescToCS is the organism's resilience to ageing‐related causes of death due to the accumulation of senescent cells (see Table 1). Extrinsic mortality was considered constant such that $p_{t}^{E}$ =1−extM, with extM being the extrinsic mortality coefficient.

Fertility *B*_*x*_ was considered as a linear function of organism age *x* such that *B*_*x*_ *= 1*−SenescRepro*.x*, where SenescRepro is the rate at which fertility declines. Then, considering an organism living in populations of infinite size in stable, homogeneous environments, its lifetime reproductive success *LRS* was:

(6) $$\mathrm{LRS}=\sum_{x=0}^{\infty }\left[\left(\prod_{t=0}^{t=x-1}p_{t}^{S}p_{t}^{C}p_{t}^{E}B_{x}\right)\right]$$

It must be stressed that if SenescRepro equals zero, then *LRS* equals life expectancy *e*_*0*_ and that cellular senescence does not participate in the fertility decline with age.

Provided that the CS probability *σ* is genetically encoded, we looked for the value σ*, which maximizes the organism *LRS* that we considered as an “optimal” value. We could therefore use this model to investigate whether CS may be naturally selected for.

For any set of parameters age‐specific, cancer incidence (taken here as equivalent to cancer mortality) $1-p_{x}^{C}$ can be calculated and the lifetime prevalence of cancer is:

(7) $${\text{prev}}^{C}=\sum_{x=0}^{\infty }\left[\left(\prod_{t=0}^{t=x-1}p_{t}^{S}p_{t}^{C}p_{t}^{E}\left(1-p_{x}^{C}\right)\right)\right]$$

### 2.2 Parameterization

To parametrize the model along a meaningful continuum of molecular, cellular, and organism parameters (*α*, *γ*, rCancerToDC, rSenescToCS, rDamageToCS) and life‐history strategies (extM and SenescRepro), we first sampled molecular, cellular, and resilience parameters in ranges corresponding to meaningful derived metrics (Table 1) in absence of extrinsic mortality and reproductive senescence. More precisely, we generated 5000 five‐uplets of input parameters (*α*, *γ*, rCancerToDC, rSenescToCS, rDamageToCS) using a Latin hypercube sampling method. All parameter values were drawn from uniform distributions whose ranges are reported in Table 2 and explained below.

**TABLE 2:** Range of input parameters defining longevity metrics in absence of accumulation of senescent cells.

Parameters/derived metrics	Maximum longevity case	Minimum longevity case
*α*	0.000497	0.006
γ	0.99	0.765
$\to e_{0}^{\text{cl}}(\alpha ,\gamma )$	2000	100
rCancerToDC	4	1
$\to e_{0}^{C}\left(\alpha ,\gamma ,\text{rCancerToDC}\right)$	2000	27
extM	0.000286	0.037
$\to e_{0}^{E}\left(\text{extM}\right)$	3500	13.5

#### 2.2.1 *α* and *γ*

In our model, the time is unitless and can be biologically interpreted as inversely proportional to the rate of damage accumulation in tissues (short times when rapid accumulation). This rate firstly depends on parameters α and *γ*. We therefore defined the time a cell line remains in an undamaged state in the absence of accumulation of senescent cells (*σ* = 0) and organism death. This time—hereafter called the *cells lines undamaged life expectancy*—can be calculated as $e_{0}^{\mathrm{cl}}\;\left(\alpha_{t},\gamma \right)=\sum_{t}^{\infty }\left(1-D_{t}\right)$. We set the ranges of parameters *α* and *γ* such that $e_{0}^{\mathrm{cl}}=100$ and $\max\;\left(e_{0}^{\mathrm{cl}}\right)=2000$ (see Table 2).

#### 2.2.2 rCancerToDC

We then calculate the *cancer‐only life expectancy* $e_{0}^{C}$(*α*, *γ*, rCancerToDC), that is the organism's life expectancy when cancer is the only mortality cause. We set the range of $e_{0}^{\mathrm{cl}}$ between 2000 and 27 (the maximum value and a quarter of the minimum value of $e_{0}^{\mathrm{cl}}$). Having a minimum value not too small allows us to explore cases where life expectancy is decreased even more by extrinsic mortality (see below).

#### 2.2.3 rSenescToCS

We then defined the differential in survival costs of accumulating damaged and senescent cells as *Δr* = rSenescToCS−rCancerToDC and set its range as [−1,4]. We could therefore sample values for rSenescToCS accordingly.

#### 2.2.4 rDamageToCS

Finally, we randomly sampled values for rDamageToCS between 1 and 10.

Once this was done, we sampled values for *extM* such that extrinsic‐mortality‐only life expectancy (Table 1) falls between $e_{0}^{E}$ = 0.5 $e_{0}^{C}$ and $e_{0}^{E}$ = 2.5 $e_{0}^{C}$ (with $e_{0}^{E}$ = 1/extM). Indeed, it made no sense to explore the parameter space where $e_{0}^{E}$ is so low that the organism is only surviving a few steps or, by contrast, where extrinsic mortality is close to zero.

Finally, we sampled the rate of reproductive senescence SenescRepro such that fertility decreased by 0% to 50% by the age of $e_{0}^{C}$.

The latin hypercube was created with the “randomLHS” function of package “lhs” (Carnell, 2022) in R 4.2.0 (R Core Team, 2020) to sample parameters within these ranges. For each of the 5000 seven‐uplets parameters (*α*, *γ*, rCancerToDC, rSenescToCS, rDamageToCS, extM, SenescRepro), the *LRS* is calculated for 1000 values of *σ* ranging from 0 to 1.

### 2.3 Statistical analyses

- Can we find parameter combinations where the accumulation of senescence cells increases the organism *LRS*? What are the drivers of such an increase and their influence on lifetime cancer prevalence?

To answer this, we first analyzed the probability to find a σ* > 0 across the explored parameter space. More precisely, we regressed the Boolean variable [σ* = 0, σ* > 0] on input parameters, their potential quadratic and cubic effects, the derived metrics (Table 2), and all two‐term interactions between derived metrics (except $e_{0}^{E}$ that is taken into account with extM), resilience parameters (rDamageToCS and rCancerToDC but not rSenescToCS as *Δr* already accounts for it) and life‐history traits (extM and SenescR). This blind approach allowed us to test for nonlinear effects and interactions between explanatory variables without having to make any biological assumptions on such effects. Analyzes were performed in *R* using the glm() function with the argument “family” set to “binomial(link=‘logit’)”. We defined the percentage gain of *LRS* as the difference between the maximum *LRS* with CS and the maximum *LRS* in the absence of CS normalized by the maximum *LRS* in the absence of CS. After selecting parameter combinations leading to a gain in *LRS* larger than 1%, we used linear regression to identify the parameters influencing the magnitude of this gain, as well as the resulting lifetime prevalence of cancer. Because of the large number of parameter combinations explored and their uniform dispersion across the parameter space, it was expected that most input parameters would significantly affect the outcomes. To assess the role played by parameters we therefore focused on the variance of the response variable explained by explanatory variables and their interactions by the mean of type I “sequential” ANOVA.

- Do these parameter combinations lead to the deceleration and decline of cancer incidence increase with age?

To analyze this, we calculated—for each parameter combinations leading to an enhanced gain in *LRS* larger than 1%—the step‐by‐step slope in cancer incidence ${h^{’}}_{t}=\left(1-p_{t+1}^{C}\right)-\left(1-p_{t}^{C}\right)$ and categorized this slope as accelerating or constant when *h*'_*t*+1_ ≥ *h*'_*t*_ > 0 (we grouped acceleration and constance as, here, we are only interested in knowing whether our model can display deceleration and decline), decelerating when 0 ≤ *h*'_*t*+1_ < *h*'_*t*_ and declining when *h*'_*t*_ < 0.

- Can we predict the effect that increasing species body size should have on lifetime cancer prevalence?

To analyze this, we made the following reasoning: (i) Although the time in our model is unitless, we can consider that—focusing for instance on mammals—the unit of time is similar between parameter combinations. In this case, increasing size decreases metabolism and cell division rate and should therefore (everything else being equal) increase $e_{0}^{\mathrm{cl}}$. (ii) Increasing size increases the number of cell lines and should therefore—in absence of cancer suppression mechanisms solving Peto's paradox—make the organism more prone to cancer for a given value of $e_{0}^{\mathrm{cl}}$. It should therefore increase the deviation of $e_{0}^{C}$ with respect to $e_{0}^{\mathrm{cl}}$. (iii) Increasing size should decrease extrinsic mortality (McCoy & Gillooly, 2008) so should increase the ratio $e_{0}^{E}/e_{0}^{C}$. (iv) Increasing size decreases the rate of reproductive senescence (Lemaître et al., 2020), so should therefore increase the ratio $\text{SenescRepro}/e_{0}^{C}$. We thus regressed the lifetime prevalence of cancer on these variables only (so blind to anything else) in the case of parameter combinations where an optimal accumulation of senescent cells is found and drew expectations on whether or not lifetime cancer prevalence is expected to increase with increasing size. Furthermore, while the potential accumulation of senescent cells has evolved *in natura*, most tests of Peto's paradox used captive data where a large part of extrinsic mortality has been removed (Vincze et al., 2022). To test the detectability of Peto's paradox in captive versus wild populations, we therefore repeated the analysis in the case of peak survival probability, i.e., when extrinsic mortality (that have been incorporated to calculate σ* as in a wild population) has been removed in the calculation of lifetime cancer prevalence (so in Equation 7, as in a “captive” population).

In case of senolytic experiments killing senescent cells at a given age, can we predict the effect on lifetime cancer prevalence and organism longevity?

To investigate this: (i) for each of the parameter combinations, we randomly sampled an age between 1 and the captive life expectancy, (ii) at this age, decreased the proportion of senescent cells by 50%, (iii) recalculated the following trajectories of senescent cells and damaged cells in the tissue, and (iv) analyzed the resulting cumulative incidence of cancer and ageing‐related causes (defined as the cumulative probability to die from, respectively, cancer or (other than cancer) ageing‐related causes, in populations where, respectively, cancer/ageing‐related causes are the only causes of mortality). Note that those calculations are done without the contribution of extrinsic mortality as senolytic experiments are carried out in controlled environments where most of the extrinsic mortality has been removed (as in captivity).

## 3 RESULTS

### 3.1 Sensitivity of σ* and gain in LRS to entry parameters

An optimal σ* > 0—so cases where the accumulation of senescent cells increased *LRS*—was found in 89% of the explored parameter space and, in 53% of all cases, *LRS* was increased by more than 1% (see Figure S1 for the complete distributions). In these cases, the mean increase in *LRS* (σ* compared with *σ* = 0) was about 3.3% (SD = 2%) and the gain in *LRS* was largely correlated to the magnitude of σ* (Pearson correlation coefficient, *⍴* = 0.44, *p* < 2.2 e‐16, Figure S1 for the complete distributions).

Table S1 shows the results of the regressions and type I ANOVAs exploring the probability of finding a σ* yielding a gain in *LRS* larger than 1%, and for those last cases, the corresponding gain in *LRS* and the lifetime cancer prevalence. The main factors explaining the probability of finding a σ* and the resulting gain in *LRS* were the magnitudes of rCancertoDC, *Δr*, and rDamageToCS, which together set the differential in survival costs of accumulating damaged and senescent cells. Hence, this result is not surprising as it is structural of the model. They together explained about 40% of the variance in the gain in *LRS*. They, however, explained only 22% of the resulting lifetime cancer prevalence. Comparatively, cellular parameters *α* and *γ* explained much less: about 4% of the variance in both the gain in *LRS* and the lifetime prevalence of cancer. The respective roles played by life‐history parameters were more contrasted. Both extrinsic mortality and senescence of reproduction played a non‐negligible role in the variance in the *LRS* gain (i.e., respectively, 19% and 25%), while extrinsic mortality was the key parameter explaining variance in lifetime cancer prevalence (about 63%).

### 3.2 Deceleration and decline in cancer incidence

**FIGURE 1:**
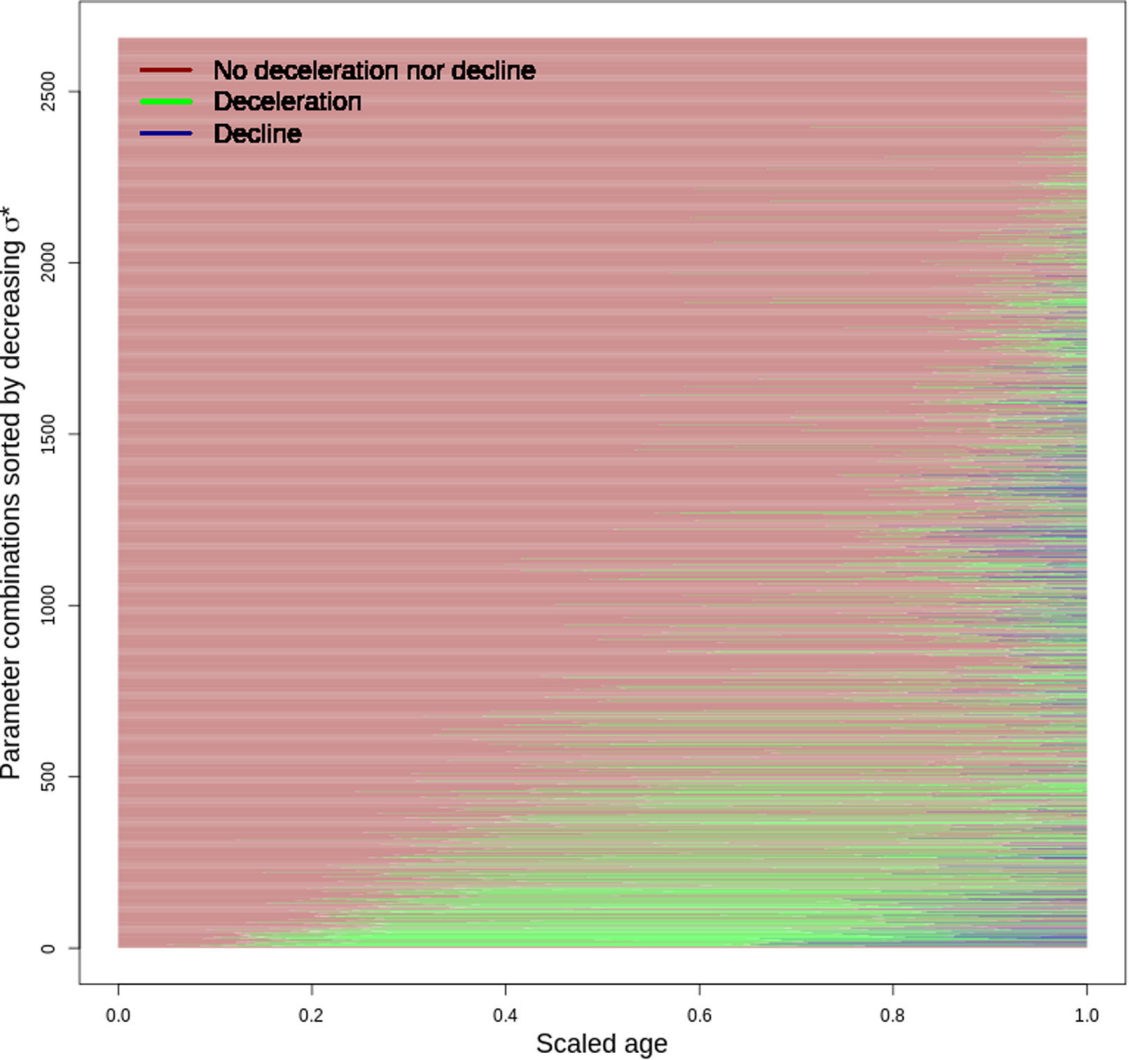
Phases of acceleration, deceleration, and decline as a function of age in simulations yielding a gain in *LRS* >1%. Figure shows that, for those simulations, deceleration and decline exist in a substantial part of the parameter space. To be contrasted with Supp. Figure 2 shows that, when CS does not allow for a gain in *LRS*, deceleration and decline are more rarely observed.

Figure 1 shows the phases of acceleration, deceleration, and decline in cancer incidence for parameter combinations leading to an increase in *LRS* of more than 1%. A deceleration is found in 33% of cases and a decline in 15% of cases. By contrast, in the simulations with a gain of *LRS* <1%, a deceleration is found in 10% of the cases and a decline in 1% (Figure S2 shows the acceleration/deceleration/decline phases in those simulations). Going back only to the simulations yielding a gain in LRS > 1%, we encoded whether a deceleration or a decline in cancer incidence was observed as a boolean variable. Table S2 gives the results of the logistic regression of that boolean variable on the parameters and derived metrics.

**FIGURE 2:**
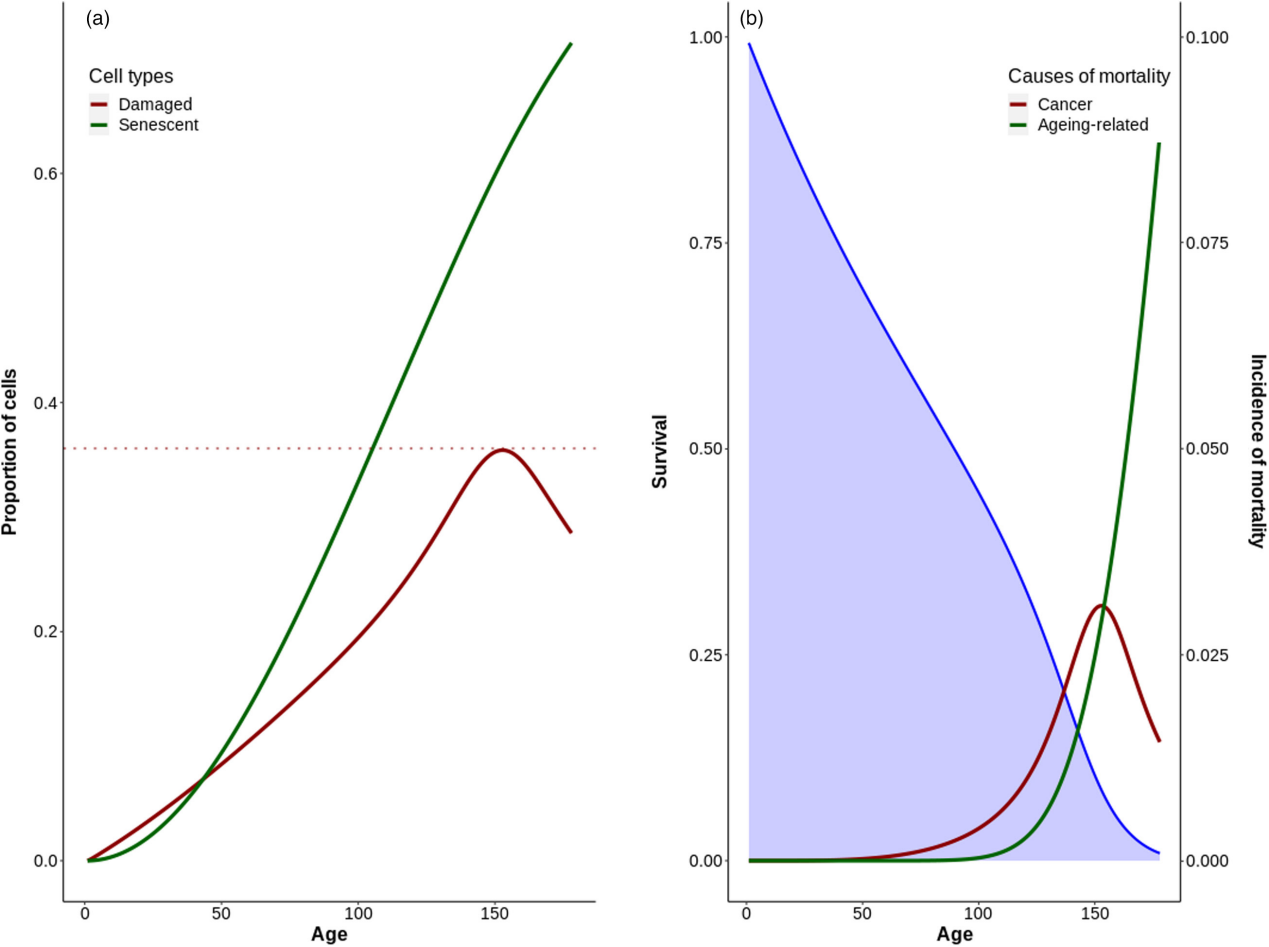
(a) Dynamics of accumulation of damaged (in red) and senescent (in green) cells. Shows the strategy of diverting a proportion of cells toward senescence up to a certain point (dashed red line). (b) Cellular dynamics translate into mortality components: Cancer (in red) and ageing‐related (green). Organismal incidence curves mirror cellular accumulation curves. Blue line depicts survival and shows that, when the decline of cancer incidence starts, survival is less than 10%.

**FIGURE 3:**
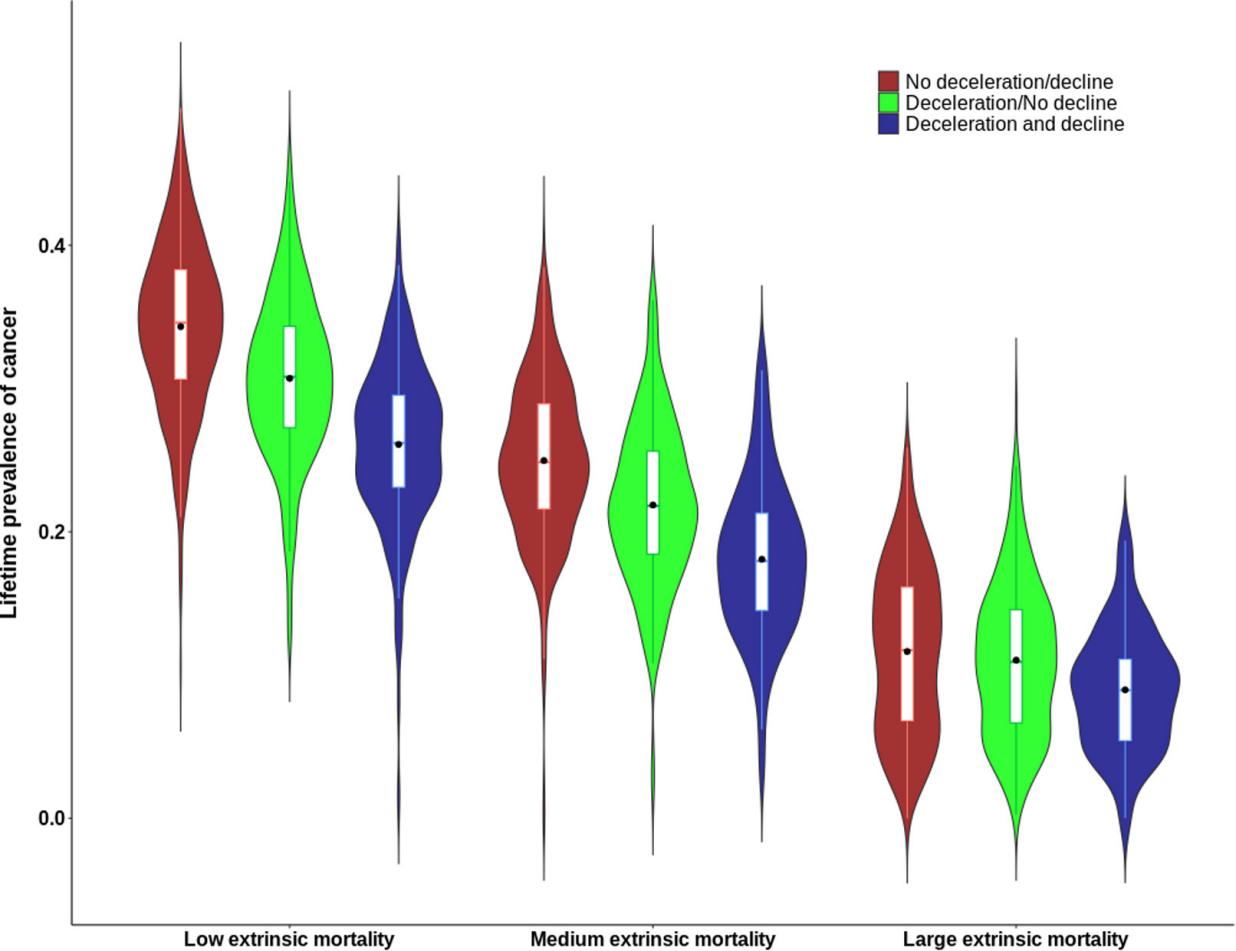
Lifetime prevalence of cancer is affected by life‐history traits (here, extrinsic mortality) and by the existence of a deceleration/decline of incidence at late ages. It shows the importance of life‐history traits when addressing whether cellular senescence evolved as an adaptation against cancer and that deceleration and decline are drivers of cancer prevalence.

A typical pattern of deceleration and decline is depicted in Figure 2, allowing to understand the mechanism behind this deceleration and decline (note that this case was chosen because the incidence of cancer and ageing‐related scaled over the same scale, but this was not always the case). In this example, the optimal strategy consisted in diverting $\left(1-\gamma \right)\;\sigma *=1\%$ of damaged cells per time step toward senescence. This optimal strategy allowed the accumulation of damaged cells early in life, leading to an early and rapid increase in cancer incidence (red line). In parallel, senescent cells accumulated at a slower pace (black line, left panel), such that mortality by other causes rises more slowly than cancers (black line, right panel). Eventually, however, when ~35% of mitotically active cells entered senescence, cell proliferation became sufficiently reduced to considerably decrease the number of new damaged cells per time step; leading to a decline in cancer incidence. Beyond this point in time, the increase in new senescent cell accumulation per time step obviously also slowed down. And yet, as the addition of even a small proportion of senescent cells considerably compromises tissue functioning and mortality by ageing‐related causes of death continued to rise (black line, right panel). Therefore, the optimal σ* stems from a balance between mortality due to cancer early in life and mortality by other ageing‐related causes later in life, with deceleration and decline in cancer incidence being mostly a byproduct. As seen in Figure 3, the effect of deceleration and decline of cancer incidence on lifetime cancer prevalence is largely influenced by the level of extrinsic mortality: when extrinsic mortality is large, nearly no difference is observed between cases with and without deceleration/decline. Together, extrinsic mortality and the deceleration/decline explained 70% of the variance in cancer incidence.

### 3.3 Effect of increasing size on lifetime prevalence of cancer

Table 3 shows the regression of the magnitude of σ* and of the lifetime prevalence of cancer in the cases of organisms “*in natura*” and in captivity on metrics linked to size. As discussed before, the main parameter influencing the magnitude of σ* was the deviation of $e_{0}^{C}$ with respect to $e_{0}^{\mathrm{cl}},\left(e_{0}^{\mathrm{cl}}-e_{0}^{C}\right)/e_{0}^{\mathrm{cl}}$. In absence of alternative cancer suppression mechanisms linked to size, an increase in size is expected to be linked to a decrease in cancer resilience, which in turn leads to selection for a larger accumulation of senescent cells translating into a negligible effect of increasing size on lifetime cancer prevalence *in natura* (Table 3). Again, lifetime cancer prevalence is mainly driven by extrinsic mortality. If our model proves true, we therefore expect large organisms to accumulate faster senescent cells (again, in the absence of or along other cancer resistance mechanisms).

**TABLE 3:** Regression of the magnitude of σ* and of the lifetime prevalence of cancer on metrics supposedly linked to size

		“In Natura” (*R* ^2^ = 81%)	“Captive” (*R* ^2^ = 40%)
Response variable	σ*	Prev.C	Prev.C
Type of regression	LM	LM	LM
Sample	Only cases where gain in *LRS* > 1%	Only cases where gain in *LRS* > 1%	Only cases where gain in *LRS* > 1%
*n*	*n* = 2656	*n* = 2656	*n* = 2656

	Coef.	Pr(>|*z*|)	Sum square, type I ANOVA	Coef.	Pr(>|*z*|)	Sum square, type I ANOVA		Coef.	Pr(>|*t*|)	Sum square, type I ANOVA	
Intercept	8.090 e‐02	***		−6.225 e‐02	***			6.290 e‐01	***		
$e_{0}^{\mathrm{cl}}$	−1.330 e‐04	***	0.846	3.115 e‐05	***	0.890		2.173 e‐04	***	3.74	
$\left(e_{0}^{\mathrm{cl}}-e_{0}^{C}\right)/e_{0}^{\mathrm{cl}}$	2.389 e‐01	***	13.789	9.899 e‐02	***	0.178		−2.005 e‐01	***	14.96	
$e_{0}^{E}/e_{0}^{C}$ ^a^	−2.325 e‐02	***	0.394	1.684 e‐01	***	21.421		6.371 e‐02	***	3.01	
$\text{SenescRepro}/e_{0}^{C}$	5.513 e+02	***	2.943	−2.770 e+02	***	0.742		−8.579 e+02	***	7.12	
Residuals			26.679			5.413				41.90	

^a^ For « captive » without extrinsic mortality, $e_{0}^{E}$ is that prior to captive condition.

In captive populations, however, $\left(e_{0}^{\mathrm{cl}}-e_{0}^{C}\right)/e_{0}^{\mathrm{cl}}$ became the main factor explaining lifetime cancer prevalence but with a negative sign: a decrease in organism resilience to cancer with size paradoxically decreased lifetime cancer prevalence. This is because $\left(e_{0}^{\mathrm{cl}}-e_{0}^{C}\right)/e_{0}^{\mathrm{cl}}$ was correlated to σ* (48%, *p* < 2.2 e‐16) leading to a larger accumulation of senescent cells and to a larger probability to observe a deceleration and decline of cancer incidence with age (Table S2). Yet, in captivity, extrinsic mortality is removed, which allows more individuals to survive up to later ages where cancer incidence decelerates and declines to the favor of other senescent‐related causes of death; decreasing the overall lifetime prevalence of cancer for large organisms. We therefore predict that, if the model is true, increased size should be linked to increased accumulation of senescent cells with age associated with a decreasing lifetime cancer prevalence with increasing size in captivity.

### 3.4 Effect of senolytic experiment on cancer risk and longevity

**FIGURE 4:**
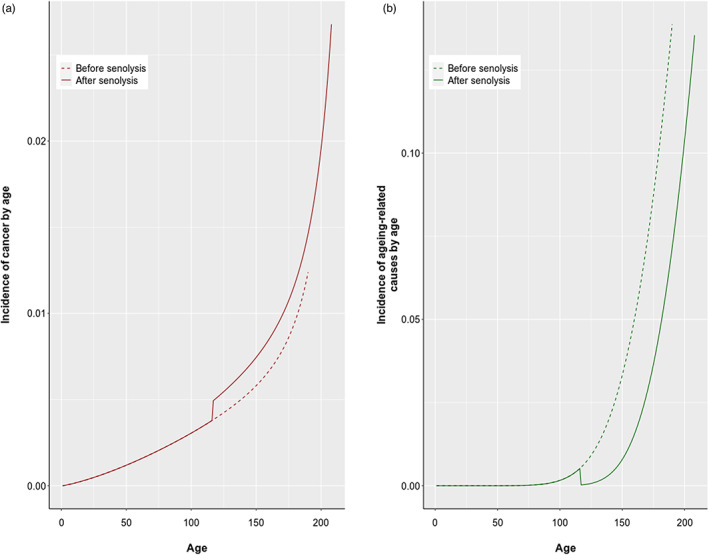
Trajectories of (a) incidence of cancer and (b) incidence of ageing‐related causes of mortality before senolysis (dashed lines) and after senolysis (solid lines). It shows that senolysis leads to an increase in cancer incidence (red) and a decrease in ageing‐related causes (green).

Results of “in silico” senolytic experiments show that killing senescent cells at any age increased the risk of cancer (in 94% of cases with a mean percentage increase of 3.9%, SD = 7.2%) and decreased the risk of other senescent‐related causes (in 97% of cases with a mean percentage decrease of 19%, SD = 25%, see Figure 4 for an example of how mortality curves for both causes are shifted after senolysis).

Overall, the increase in cancer mortality was more moderate with only 12% of simulations with more than 10% increase as opposed to (other) ageing‐related causes where 43% of cases exhibited a decrease superior to 10%. Both variables were not significantly correlated (*ρ* = 0.098, *p* > 0.05), suggesting that different parameters and model properties may have influenced changes in the cumulative incidence of both causes. Indeed, linear regressions of both variables on all the parameters show that many terms are significantly associated (Table S3), but the exhaustive analysis of those models is out of the scope of this paper.

**FIGURE 5:**
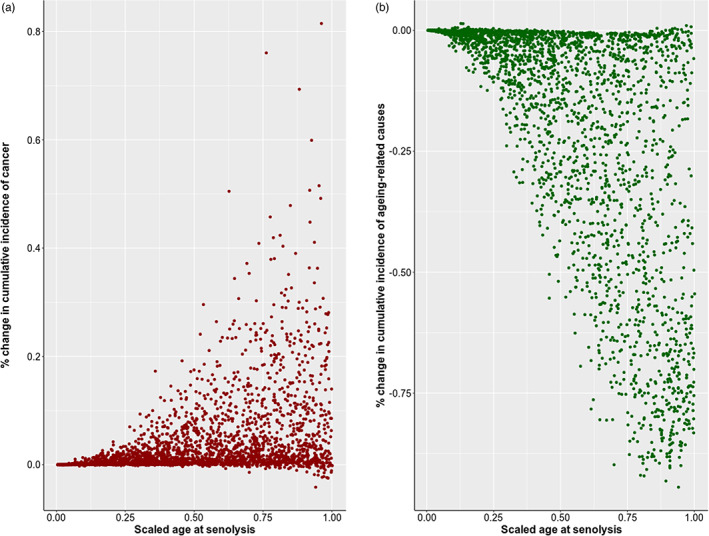
Percentage change in the cumulative incidence of (a) cancer and (b) ageing‐related causes after senolysis.

As a preliminary analysis, though, we tested the correlations between both changes in cumulative incidences and (i) the age at senolysis and (ii) the optimal σ*. Regarding age at senolysis, only the percentage change in the cumulative incidence of (other) senescent‐related causes was significantly negatively correlated with age at senolysis (*ρ* = −0.63, *p* < 0.05) whereas the percentage change in the cumulative incidence of cancer is nonsignificantly correlated (*p* > 0.05). As seen in Figure 5 (panels (a) and (b)), the later the age at senolysis, the more accentuated the differences in the cumulative incidence of both causes were. The reason is the following. It is the entrance into senescence that prevents cancer. However, in our model, killing senescent cells induces compensatory proliferation after senolysis, which, in turn, briskly accelerates damage accumulation and increases cancer risk (see Figure 5). The later the senolysis, the larger the proportion of senolysed‐senescent cells replaced by damaged cells, and the larger this effect.

Regarding the optimal σ*, the percentage change in cancer cumulative incidence (*ρ* = 0.28) and percentage change in (other) senescent‐related causes (*ρ* = 0.24) are significantly but weakly correlated. This lack of correlation stems from the fact that, for a given value of σ*, very diverse values of changes in cumulative incidence are found. Once again, further analysis of other confounders is needed to understand the effect.

## 4 DISCUSSION

In this study, we explored the hypothesis that cellular senescence can be an adaptive strategy by balancing two intrinsic mortality components, that is cancer and other ageing‐related causes of death. We do this using a theoretical model that first describes the dynamics of senescent and damaged cell accumulation with age. Cells are described by three key features: a probability to get damaged and, once damaged, a probability to die from apoptosis and a probability to enter senescence. Accumulation of damaged cells leads to an increased risk of developing cancer whereas the accumulation of senescent cells increases the risk of developing (other than cancer) ageing‐related diseases. Both risk increases are modulated by parameters setting the organism's resilience to, respectively, ageing‐related causes and cancer. Lastly, we use the “Lifetime Reproductive Success” (*LRS*), also incorporating extrinsic mortality and reproductive senescence, as a metric to test our hypothesis and explore different questions.

First, we assess whether an optimal probability (in the sense that it optimizes *LRS*) for a damaged cell to enter senescence can be found. We find that such probability (denoted σ*) exists in a large majority of our explored parameter space, with half of this space allowing for a gain in *LRS* larger than 1%. We found that the likelihood to find σ* yielding a gain of *LRS* larger than 1% is first influenced by the parameters modulating the dynamics of damage within tissues and the organism's resilience to both intrinsic causes of mortality. A key perspective for empirical studies investigating our hypothesis is therefore to better investigate these dynamics with age and how they translate into specific mortality components. Beyond cellular parameters, we find that life‐history parameters, here extrinsic mortality and reproductive senescence, also play a major role: alongside cellular parameters, they have a key influence on the extent of the *LRS* gain (Table S1). It makes sense from an evolutionary point of view as a strategy balancing the accumulation of senescent cells should have jointly evolved with life‐history features such as size and position along the slow‐fast continuum (Cayuela et al., 2020). Similarly, we find that the lifetime cancer prevalence is mainly influenced by life‐history traits such that this metric is a poor indicator of the underlying molecular and cellular processes. For studying ageing, one should therefore go beyond prevalence and focus on age‐specific cancer incidence (see also Ritter et al. (2003) on the comparative use of mortality and incidence in the Doll‐Armitage model).

After finding that some σ* values can maximize an organism's *LRS*, we tried to see whether such strategy could explain a deceleration and/or decline of the individuals' cancer risk with age as an alternative to population effects due to heterogeneity in cancer risks (Anisimov et al., 2005). The answer is yes: a substantial proportion of the parameter space allows for some deceleration and decline. Mechanistically, this is due to a reduced pool of mitotically active cells leading to a slowdown of damaged cell accumulation. Moreover, when examining those combinations, the probability to observe a deceleration/decline is a function of molecular, cellular, and resilience parameters including σ* (Figure 1 and Figure S2). We further show that deceleration/decline of cancer incidence interacts with extrinsic mortality in shaping lifetime cancer prevalence. As a result, a large part of the variance of the lifetime prevalence of cancer is driven by the existence of deceleration or decline, which is driven by σ*, which is itself driven by the extent of extrinsic mortality. This emphasizes the fact that, again, trade‐offs between mortality components have jointly evolved under complex age‐specific evolutionary pressures across different levels of interaction from cells to the environment. Lastly, it is important to keep in mind that the late‐life deceleration/decline is explained—in our model—as a byproduct of two functions (cancer and ageing‐related cause incidences) that increase early in life. Therefore, although deceleration and decline are important drivers of lifetime cancer prevalence, they are nevertheless the byproduct of the optimization, earlier in life, of the two increasing functions.

The role of extrinsic mortality in the previous results is not trivial as the role of life‐history traits is central to the third question we ask: to what extent size influences the resulting lifetime cancer prevalence? Results first provide a prediction: large organisms should accumulate more senescent cells and, in captivity, exhibit a reduced lifetime prevalence of cancer (due to a late deceleration/decline). It is important to bear in mind that this prediction holds when there are no additional cancer resistance mechanisms that evolved concomitantly with larger sizes/longer lifespans, though a recent study suggested that long‐lived mammals indeed evolved higher CS rates than shorter‐lived ones (Attaallah et al., 2020). We also find that the effect of size would be different in wild and in captive populations. Indeed, captive populations do not suffer from extrinsic mortality (or, at least, extremely reduced levels) and individuals can therefore leave long enough (i) to get cancer and (ii) so that deceleration and decline is observable. Those results suggest that Peto's paradox might not be readily observable in wild populations and illustrate that the lifetime prevalence of cancer is not a trivial metric, but rather a combination of different aspects (size, captive vs. wild, life‐history traits…). Overall, those results call for a large survey on the pattern of cancer incidence with age in captive animal populations to check whether cancer incidence deceleration is observed.

Finally, we sought to assess whether our model recapitulates results found in senolytic experiments. Results are contrasted. On the one hand, similarly to senolytic experiments (Baker et al., 2011, 2016), we find that killing senescent cells quasi‐systematically decreases the cumulative incidence of senescent‐related mortality. This effect is modulated by age at senolysis: the later the age at senolysis, the larger the decrease in cumulative incidence. It would be interesting to reiterate senolytic experiments with the same modified mice model as previously described (Baker et al., 2016) to assess whether this is the same in vivo. On the other hand, we find—even if more moderate and not systematic—an increase in cancer cumulative incidence, whereas, in murine models, senolysis delayed tumorigenesis and incidence is unchanged (Baker et al., 2011, 2016). The reason for this can be three‐fold: (i) in our model, senolysis is followed by compensatory proliferation. A proxy for the onset of senescence can be weight loss (Richardson et al., 2014) as loss can reflect the moment where homeostatic proliferation does not compensate for cellular senescence‐induced loss of tissue function. In mice models, the animal weights are not reported and weight changes paint different pictures in different organs (see Supplementary Material in Baker et al. (2016)) so we do not know whether such proliferation is also observed, (ii) mice are not a relevant model to assess whether senolysis indeed increases cancer incidence because, even with delayed ageing, their lifespan is limited. If doable, repeating senolytic experiments on more long‐lived mammals would bring useful insights into the effect of senolysis on the risk of developing cancer later on. Finally, (iii) the additional proportion of damaged cells due to CS is insufficient to depict the actual loss of tissue connectivity and inflammatory influence of the SASP (the senescent secretome; see Coppé et al., 2010 or Campisi & Robert, 2014). Yet, it is possible that the empirical benefit of senolysis on cancer incidence is only related to the suppression of the SASP. In order to test this hypothesis, senolytic experiments could be performed with and without pharmacological control of the SASP (Malaquin et al., 2016).

Overall, our model builds on knowledge from cellular experimental observations and allows us to draw predictions under our hypothesis. It also shows that the deceleration and decline of cancer incidence could be the results of mitotically active cell exhaustion, as suggested by other works (Tomasetti et al. (2019), considering stem cells). Although simple, this model also allows us to bridge cellular dynamics and their organismal consequences. With that in hand, we show the importance of life‐history traits in shaping the evolution of a species‐specific risk of cancer.

Despite being adapted to address our questions, our model remains simple. First, we model the damage level within tissue and single cells cannot accumulate damage. But we know this is largely not true: cells inherit their mutation burden from their mother. Hence, the sequence of mutations required to turn a cell into a carcinogenic one can only be acquired along a lineage. A perspective of this work would therefore be the exploration of the same cellular dynamics in a tissue structured into lineages. Moreover, one of the current limitations of in vivo approaches is that they lose the architecture of the tissue (van Deursen, 2019), rendering impossible investigation on potential clusters of senescent cells (see Privman et al., 2016 and Gorshkov et al., 2016) and the cellular communication that takes place within such clusters through the *SASP* (senescent secretome). This is one more argument advocating for a more refined model taking into account the way dynamics are intertwined with tissue spatialisation, anatomy/histology, and paracrine effects of senescent cells. Furthermore, such a model would allow us to differentiate between replicative senescence (induced by telomere attrition) and stress‐induced senescence (induced by DNA damage) as both have been shown to lead to different secretory phenotypes (Wissler Gerdes et al., 2020) potentially influencing cancer risk differently. One last simplified cellular aspect of our model is the number of processes that are taken into account here. First, we only consider mutations whereas epimutations have been shown to play a role in carcinogenesis (Yang et al., 2022) and to accumulate with age (Pal & Tyler, 2016). In our model, we consider mutations and epimutations altogether as potential “damage”. The difference between genetic and epigenetic alterations is two‐fold: (i) epigenetic alterations are reversible (thus, their transmission from mother to daughter cells is not guaranteed) and (ii) it is unknown whether epimutations alone can trigger either apoptosis or cellular senescence. Including epimutations in our model would suppose contrasting different scenarios assuming different roles of epimutation in the carcinogenesis risk. Second, we consider a limited number of processes involved in tissue homeostasis, but others exist such as cellular quiescence (a reversible cell cycle arrest) or autophagy (a degradation process triggered by metabolic stress). Autophagy is out of the scope of that paper as we do not explicitly model metabolism. Regarding cellular quiescence, we can hypothesize the influence it would have on the presented results. If the exit of quiescence is conditioned by the repair of damage (Beerman et al., 2014), then implementing quiescence in our model would result in increasing the pool of healthy cells without compensatory divisions. If exit of quiescence is possible without the damage being repaired, then the pool of damaged cells would periodically (and independently of division) increase. In our model, we argue that cellular quiescence would mainly change the shape and pace of damage accumulation but not the overall results. One other simplified aspect of the model is how we considered the life‐history trait relationship with age: we considered reproductive senescence but not as a function of cellular senescence. Yet, part of the age‐dependent decline in fertility could be linked to CS through the exhaustion of cell progenitors. It would therefore be interesting to re‐calculate the *LRS* in a scenario where CS scales the reproductive senescence. All the above limitations lead to downscaling perspectives, i.e., refining our approach to cellular processes. Another perspective is actually to upscale the model: here, we modeled deterministic phenomenons but, by introducing stochasticity in the model, it could be used to generate populations of heterogeneous individuals (regarding competing mortality risks). Such a model would allow us to test the hypothesis of selective disappearance (see Section 1: Introduction) and would be of interest to the epidemiological field.

Finally, it is important to note that other models of carcinogenesis exist. For instance, the initiation‐progression model posits that mutations are needed for cells to progress toward carcinogenicity but that carcinogenesis itself is triggered by exposure to intrinsic and extrinsic factors that allow clonal expansion of damaged lineages. Our model is not adapted to incorporate clonal expansion as we do not take into account the selective value of damage and accumulation within lineages (hence, we are blind to tumor heterogeneity). Regarding the potentiation of damage by exposure to factors, we argue that our model assumes extrinsic factors to be constant but that it could be modified to take into account intrinsic factors with age‐dependent variability. First, to account for immune senescence, rCancerToDC could be a globally decreasing function of age. The precise shape of that function, e.g., linear, sigmoid etc., would, however, require further assumptions. In that case, we would furthermore expect a selection for larger values of σ* as more CS would be needed to account for the decrease in resilience to damage with age. Additionally, we only consider the probability for a cell to get damaged (*α*) and the fertility (through the parameter SenescRepro) to be subject to physiological senescence, but the model could be extended to account for age‐dependent variability of all processes (e.g., the probability to undergo apoptosis, a decrease in the resilience to the accumulation of senescent cells etc.).

Besides these limitations that pertain to the model type we use and to the theoretical framework we operate in, we show that under a simple set of equations, the hypothesis that cellular senescence is adaptive could be validated and that predictions for further empirical studies can be drawn. This work is therefore important in the context of cellular biology where this hypothesis has been around for some time without (to our knowledge) having been tested formally. Furthermore, our framework shows the vital importance of integrating knowledge from cells to organisms (ecology and evolution) to complete the cancer puzzle.

## 5 CONFLICTS OF INTEREST

None.

## Supporting information


Figures S1‐S2

Tables S1‐S3

## Data Availability

All scripts will be available on a Github repository.
