# Supplementary material for: Modeling of senescent cell dynamics predicts a late‐life decrease in cancer incidence
Source: Evol Appl. 2023 Mar 1;16(3):609–24. doi: 10.1111/eva.13514 (PMC10033854; doi:10.1111/eva.13514)
Supplement: Supplementary file 1 — Figures S1‐S2 [file EVA-16-609-s002.docx]

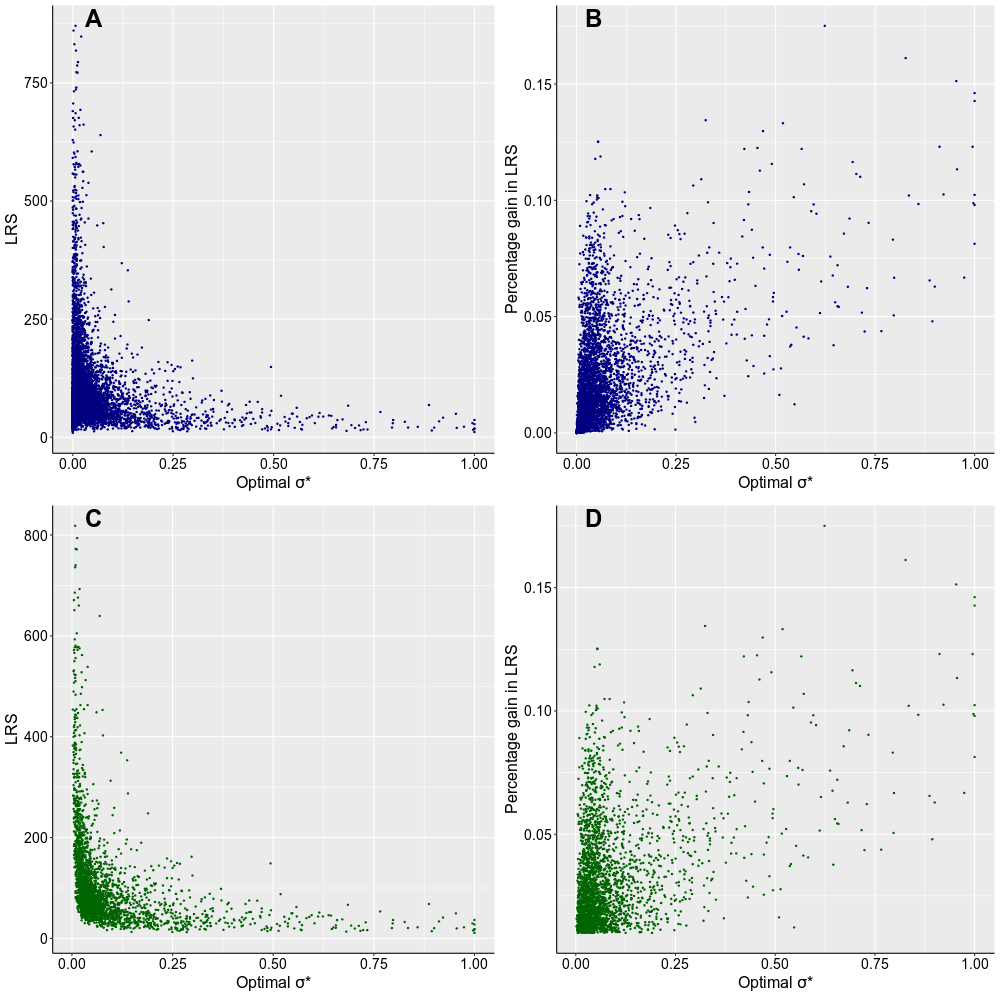


Supplementary Figure 1 : panels A and B show the distributions of *LRS* (green) and percentage gain in *LRS* (blue) in all simulations. Panels C and D show the distributions of *LRS* (green) and percentage gain in *LRS* in the simulations where *σ** allows for a gain in LRS > 1%


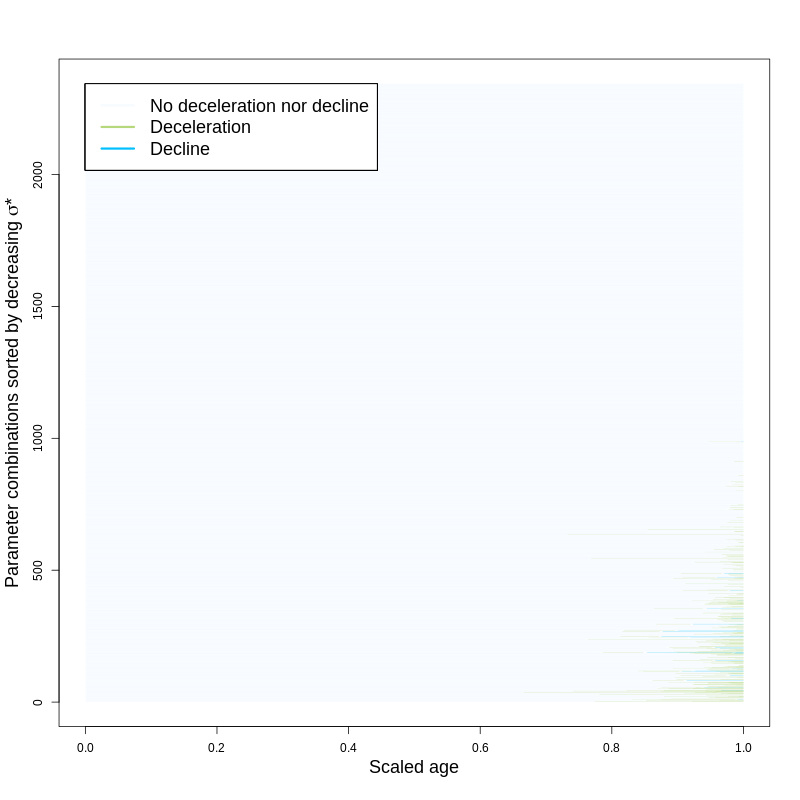


Supplementary Figure 2: Phases of acceleration, deceleration and decline as a function of age in simulations yielding a gain in *LRS* < 1%. Figure shows that, for those simulations, deceleration and decline rarely exist and therefore that deceleration and decline are more associated with values of *σ** leading to a gain in fitness (i.e when cellular senescence can be considered as adaptive). To be contrasted with Figure 1
