# Supplementary material for: Modeling of senescent cell dynamics predicts a late‐life decrease in cancer incidence
Source: Evol Appl. 2023 Mar 1;16(3):609–24. doi: 10.1111/eva.13514 (PMC10033854; doi:10.1111/eva.13514)
Supplement: Supplementary file 2 — Tables S1‐S3 [file EVA-16-609-s001.pdf]

	Can we find scenarios where the accumulation of senescent cells is an optimal stragegy?			What are the drivers of the gain in fitness of these scenarios?			What are the drivers on the prevalence of cancer of such scenarios?		
Response variable	Boolean (1/0). σ* leads to a gain in *LRS*> 1%			log(Gain in fitness)			Prevalence of cancer		
Type of regression	GLM - Binomial (logit)			LM			LM		
Sample	All scenarios			Only scenarios where gain in *LRS*>1%			Only scenarios where gain in *LRS*>1%		
n	n=5000			n=2656			n=2656		
	Coef.	Pr(>|z|)	Sum square, type I anova	Coeff.	Pr(>|t|)	Sum square, type I anova	Coef.	Pr(>|t|)	Sum square, type I anova
α	-2.963e+03	.	9.3	3.770e+02	***	14.30	1.232e+01		1.009
α^2	8.248e+05	*	0.4	-5.160e+04	*	0.51	-2.829e+03		0.017
α^3	-6.964e+07	*	0.2	3.651e+06	.	1.10	2.027e+05		0.005
γ	-2.293e+02		48.4	-1.427e+01		22.08	-1.652e+01	*	0.258
γ^2	3.509e+02		3.0	2.573e+01		3.80	1.947e+01	*	0.001
γ^3	-1.782e+02		0.5	-1.537e+01		0.03	-7.602e+00	*	0.002
e_0^{cl}	-9.734e-03		0.2	-9.663e-04	*	0.21	1.388e-04	*	0.020
rCancerToDC	-1.147e+01	***	0.3	-4.129e+00	***	20.25	3.018e-01	***	0.284
r.CancerToDC^2	5.874e+00	***	4.1	1.631e+00	***	63.69	-1.492e-01	***	0.298
r.CancerToDC^3	-6.875e-01	***	0.4	-1.838e-01	***	16.48	1.911e-02	***	0.201
e_0^C	6.213e-03		0.0	1.398e-03	**	0.16	-2.661e-04	***	0.024
Δr	1.175e+01	***	448.4	1.302e+00	***	193.29	-7.935e-02	***	3.308
Δr^2	-1.292e+00	***	18.3	-1.269e-01	***	0.24	4.919e-03	*	0.037
Δr^3	1.010e-01	**	7.5	5.034e-03	*	0.10	1.376e-04		0.054
rDamageToCS	7.817e+00	***	27.2	6.186e-01	***	20.65	-4.495e-02	***	0.855
rDamageToSC^2	-1.255e+00	***	17.4	-8.890e-02	***	10.44	5.924e-03	***	0.448
rDamageToSC^3	6.413e-02	***	7.4	4.234e-03	***	5.12	-2.585e-04	***	0.002
extM	-8.435e+01		39.9	-7.496e+01	***	104.45	-2.407e+01	***	9.845
extM^2	3.413e+03		17.3	2.688e+03	***	45.88	7.393e+02	***	4.567
extM^3	-5.702e+03		3.7	-2.669e+04	***	7.31	-7.876e+03	***	1.849
SenescRepro	2.285e+03	***	34.5	1.177e+02	***	143.98	2.526e+01	***	0.306
SenescRepro^2	-2.353e+05	***	11.2	-1.145e+04	***	39.89	-1.547e+03	***	0.005
SenescRepro^3	8.502e+06	***	2.9	4.297e+05	***	5.05	3.443e+04	***	0.002
$\Delta r * e_0^{cl}$	7.431e-03	**	0.1	3.451e-04	**	0.08	-6.154e-05	***	0.003
$\Delta r * e_0^C$	-6.841e-03	**	17.4	-3.011e-04	*	28.08	8.707e-05	***	0.354
Δr**r.CancerToDC*	-2.332e+00	***	19.9	-1.973e-01	***	28.83	1.358e-02	***	0.217
Δr**rDamageToCS*	1.730e-01	***	5.5	9.593e-03	***	0.72	-1.151e-03	***	0.014
*rDamageToCS**e_0^{cl}	-2.730e-03	***	0.1	-2.515e-04	***	0.00	3.625e-05	***	0.014
*rDamageToCS**e_0^C	2.399e-03	***	3.5	2.149e-04	***	3.66	-4.134e-05	***	0.059
*extM**e_0^{cl}	-6.627e-01	.	0.0	5.455e-02	**	0.84	1.026e-02	***	0.021
*extM**e_0^C	-5.826e+00	***	9.5	-7.325e-01	***	29.46	-2.256e-01	***	1.223
*extM**Δr	1.272e+01		0.2	-9.559e-01		0.00	1.237e+00	***	0.064
*extM*rDamageToCS*	-5.306e+00		0.5	-1.465e+00	***	1.81	2.870e-01	***	0.044
*SenescRepro**e_0^{cl}	3.997e+00	***	0.5	6.292e-01	***	0.19	-3.654e-02	***	0.111
*SenescRepro**e_0^C	-3.061e+01	***	27.2	-3.634e+00	***	56.19	-9.066e-02	***	0.043
*SenescRepro**Δr	-7.754e+00		0.3	-2.157e+00		0.04	-3.717e+00	***	0.147
*SenescRepro*rDamageToCS*	-6.198e+01	***	1.5	-4.471e+00	***	1.47	-4.295e-01	***	0.014
Residuals			456.4			116.36			2.921
R^2	79% (MacFadden R^2)			88%			90%		

Supplementary Table 1: Regression models for the first question of the paper. Regression coefficients are reported along with the significance of the p-values and the sums of squares associated with each variable (type

	Can we find scenarios where accumulation of senescent cells leads to deceleration and decline in cancer incidence at old ages?		
Response variable	Boolean (0/1) if a deceleration or a decline is observed		
Type of regression	GLM - Binomial (logit)		
Sample	Only scenarios where gain in *LRS*>1%		
n	n=2656		
	Coef.	Pr(>|z|)	Sum square, type I anova
σ^*	-8.816e+00	***	32.0
α	-1.859e+03	***	25.6
α^2	5.022e+05	***	1.5
α^3	-4.322e+07		0.3
γ	4.867e+03	***	1.4
γ^2	-5.880e+03	***	3.1
γ^3	2.372e+03		0.2
e_0^{cl}	-4.206e-02		0.2
rCancerToDC	9.342e+00	***	72.0
$rCancerToDC^2$	-6.924e-01	***	24.5
$rCancerToDC^3$	1.295e-01	***	6.8
e_0^C	-2.987e-02	***	41.9
Δr	4.085e+00	***	64.9
Δr^2	-7.844e-01	***	4.5
Δr^3	3.052e-02		0.3
rDamageToCS	1.674e+00	***	5.0
rDamageToCS^2	-2.806e-01	***	2.6
rDamageToCS^3	1.258e-02	**	1.2
extM	4.331e+02		0.3
$extM^2$	-1.488e+04	***	5.9
$extM^3$	1.266e+05	***	2.0
SenescRepro	-4.720e+01	***	5.6
$SenescRepro^2$	2.170e+04	.	0.5
$SenescRepro^3$	-7.827e+05	***	1.8
$\Delta r^* e_0^{cl}$	9.399e-03		0.1
$\Delta r^* e_0^C$	-6.639e-04		0.0
Δr*rCancerToDC*	-3.232e-02	**	1.0
Δr*rDamageToCS	-4.929e-02	**	0.8
$rDamageToCS^* e_0^{cl}$	4.985e-03		0.1
$rDamageToCS^* e_0^C$	-3.219e-03	*	0.6
$extM^* e_0^{cl}$	8.743e+00	***	6.1
$extM^* e_0^C$	-1.908e+01	***	29.9
$extM^*\Delta r$	4.991e+01		0.0
*extM*rDamageToCS*	2.131e+01	*	0.8
$SenescRepro^* e_0^{cl}$	8.697e+00		0.0
$SenescRepro^* e_0^C$	1.158e+01	***	1.6
$SenescRepro^*\Delta r$	1.853e+01		0.1
*SenescRepro*rDamageToCS*	-5.382e-01		0.1
Residuals			317.2
R^2	64% (MacFadden R^2)		

Supplementary Table 2: GLM for the second question of the paper. Regression coefficients are reported along with the significance of the p-values and the sums of squares associated with each variable (type I ANOVA).

	In case of senolytic experiments killing senescent cells at a given age, can we predict the effect on lifetime cancer prevalence and organism longevity?			
Response variable	Percentage change in Cumulative Incidence of cancer after senolysis		Percentage change in Cumulative Incidence of ageing-related causes after senolysis	
Type of regression	LM			
Sample	Only scenarios where gain in *LRS* > 1%			
n	n=2651		n=2656	
	Coef.	Pr(>|z|)	Coef.	Pr(>|z|)
Intercept	1.02E+01	***	4.19E+00	
σ	4.06E-01	.	-1.84E+00	***
Age.senolyse	6.22E-04	***	-4.68E-04	
Alpha	9.73E+01	***	9.61E+01	**
Alpha^2	7.88E+02		1.31E+04	*
Alpha^3	-1.44E+05		-1.07E+06	*
Gamma	-3.73E+01	***	-1.95E+01	
Gamma^2	4.37E+01	***	2.53E+01	
Gamma^3	-1.64E+01	***	-1.01E+01	
rCancerToDC	1.24E-01	*	2.17E-01	.
rCancerToDC^2	-7.80E-02	***	-1.04E-01	**
rCancerToDC^3	1.02E-02	***	1.54E-02	***
rSenescToCS	1.57E-01	***	3.68E-01	***
rSenescToCS^2	7.81E-03		-1.80E-03	
rSenescToCS^3	5.58E-05		5.79E-04	
rDamageToCS	2.17E-02	**	3.03E-02	.
rDamageToCS^2	-1.87E-03	.	-5.15E-03	*
rDamageToCS^3	1.01E-04		2.44E-04	.
SenescRepro	8.81E+00		1.90E+01	
SenescRepro^2	1.20E+03	***	5.97E+02	
SenescRepro^3	-2.90E+04	**	-1.96E+04	
extM	-1.23E+01	**	-2.03E-01	
extM^2	2.26E+02	***	9.08E+01	
extM^3	-2.37E+03	***	-1.00E+03	
*Alpha*Gamma*	-1.33E+02	***	-1.19E+02	***
*Alpha*rCancerToDC*	1.32E+00		-1.53E+01	***
*Alpha*rSenescToCS*	5.31E+00	***	6.41E+00	***
*Alpha*rDamageToCS*	2.47E-01		-3.25E-01	
*Alpha*SenescRepro*	2.06E+02		-9.87E+02	
*Alpha*extM*	-2.77E+02		-5.05E+02	
*Gamma*rCancerToDC*	1.45E-01	***	8.98E-02	
*Gamma*rSenescToCS*	-2.74E-01	***	-4.11E-01	***
*Gamma*rDamageToCS*	-1.39E-02	*	6.73E-03	
*Gamma*SenescRepro*	-3.00E+01	**	-2.47E+01	
*Gamma*extM*	9.20E+00	*	-2.69E+00	
*rCancerToDC*rSenescToCS*	-9.32E-03	***	-1.42E-02	***
*rCancerToDC*rDamageToCS*	-1.20E-03	*	-3.38E-04	
*rCancerToDC*SenescRepro*	-7.02E+00	***	-1.65E+00	
*rCancerToDC*extM*	-8.33E-01	*	1.22E+00	
*rSenescToCS*rDamageToCS*	7.97E-04	*	2.98E-04	
*rSenescToCS*SenescRepro*	4.06E+00	***	-1.04E+00	
*rSenescToCS*extM*	1.73E-01		-1.04E+00	**
*rDamageToCS*SenescRepro*	1.85E-01		-2.47E-01	
*rDamageToCS*extM*	-8.37E-02		-2.92E-02	
*SenescRepro*extM*	3.84E+01		9.67E+01	
*σ*Age.senolyse*	2.17E-03	***	3.44E-03	***
*σ*Alpha*	3.95E+01	***	5.67E+01	**
*σ*Gamma*	-2.04E-01		1.24E+00	**
*σ*rCancerToDC*	1.24E-03		5.04E-01	***
*σ*rSenescToCS*	-2.38E-02		-2.80E-02	
*σ*rDamageToCS*	-4.57E-03		-2.38E-03	
*σ*SenescRepro*	-1.81E+01	***	-4.15E+00	
*σ*extM*	-3.14E+00	*	3.75E+00	
*Age.senolyse*Alpha*	-1.43E-01	***	-1.27E+00	***
*Age.senolyse*Gamma*	-1.00E-03	***	8.10E-04	*
*Age.senolyse*rCancerToDC*	-3.23E-05	*	-1.49E-04	***
*Age.senolyse*rSenescToCS*	6.02E-05	***	-1.04E-05	
*Age.senolyse*rDamageToCS*	1.86E-06		1.10E-06	
*Age.senolyse*SenescRepro*	1.80E-01	***	2.31E-01	***
*Age.senolyse*extM*	6.28E-02	***	7.67E-02	***
Adj. R2	0.7004		0.8958	

Supplementary Table 3: LM for the fourth question of the paper. Regression coefficients are reported along with the significance of the p-values.
